# Genetic analysis of nonalcoholic fatty liver disease within a Caribbean–Hispanic population

**DOI:** 10.1002/mgg3.168

**Published:** 2015-08-11

**Authors:** Deborah Edelman, Harmit Kalia, Maria Delio, Mustafa Alani, Karthik Krishnamurthy, Mortadha Abd, Adam Auton, Tao Wang, Allan W. Wolkoff, Bernice E. Morrow

**Affiliations:** ^1^ Department of GeneticsAlbert Einstein College of Medicine1301 Morris Park Ave.BronxNew York10461; ^2^ Division of Gastroenterology and Liver DiseasesMontefiore Medical Center and Albert Einstein College of MedicineBronxNew York10461; ^3^ Marion Bessin Liver Research CenterAlbert Einstein College of MedicineBronxNew York10461; ^4^ Department of Epidemiology & Population HealthAlbert Einstein College of MedicineBronxNew York10461; ^5^ Department of Anatomy and Structural BiologyAlbert Einstein College of MedicineBronxNew York10461

**Keywords:** ABCC2, CHUK, ENPP1, ERLIN1, fatty Liver, gene, hispanic population, liver genetics, NAFLD, NASH, nonalcoholic fatty liver disease, nonalcoholic steatohepatitis, PNPLA3, polymorphism, SAMM50.

## Abstract

We explored potential genetic risk factors implicated in nonalcoholic fatty liver disease (NAFLD) within a Caribbean–Hispanic population in New York City. A total of 316 individuals including 40 subjects with biopsy‐proven NAFLD, 24 ethnically matched non‐NAFLD controls, and a 252 ethnically mixed random sampling of Bronx County, New York were analyzed. Genotype analysis was performed to determine allelic frequencies of 74 known single‐nucleotide polymorphisms (SNPs) associated with NAFLD risk based on previous genome‐wide association study (GWAS) and candidate gene studies. Additionally, the entire coding region of *PNPLA3,* a gene showing the strongest association to NAFLD was subjected to Sanger sequencing. Results suggest that both rare and common DNA variations in *PNPLA3* and *SAMM50* may be correlated with NAFLD in this small population study, while common DNA variations in *CHUK* and *ERLIN1,* may have a protective interaction. Common SNPs in *ENPP1* and *ABCC2* have suggestive association with fatty liver, but with less compelling significance. In conclusion, Hispanic patients of Caribbean ancestry may have different interactions with NAFLD genetic modifiers; therefore, further investigation with a larger sample size, into this Caribbean–Hispanic population is warranted.

## Introduction

Nonalcoholic fatty liver disease (NAFLD) has become the most common cause of chronic liver disease in western countries, with a prevalence of approximately 30% in the United States (Lazo et al. 2011). It is characterized by excessive fat accumulation in the liver, or hepatic steatosis. The clinical significance of steatosis is correlated with its histological severity. Nonalcoholic fatty liver disease can progress to a more severe disorder. The disease can range from simple steatosis to steatosis with inflammation (nonalcoholic steatohepatitis or NASH), fibrosis, and cirrhosis (Adams et al. 2005). A total of 2.9–20% of NAFLD patients will develop NASH dependent on the disease lead‐time (Charlton et al. 2001; Lebovics and Rubin 2011). Once NASH develops, an estimated 17–49% of cases progress to cirrhosis and, once present, patients are at risk for liver decompensation and hepatocellular carcinoma (Bugianesi et al. 2002; Ratziu et al. 2002; Argo et al. 2009). Overall, a small percentage of individuals with NAFLD progress to end‐stage cirrhosis or liver cancer. However, given the total number of cases of NAFLD, this still amounts to a large number of patients suffering from these significant morbidities.

Nonalcoholic fatty liver disease is frequently associated with several coexisting conditions including obesity, visceral fat, hyperlipidemia, and type 2 diabetes (Angulo 2002). Diabetes is not only a risk factor for NAFLD independent of obesity (Gupte et al. 2004), its presence also seems to compound the risk of histopathologic progression of steatosis among obese patients (Silverman et al. 1990). This logically follows as NAFLD is closely associated with insulin resistance, although the direction of this association has not been established (Marchesini et al. 1999; Angulo 2002; Paschos and Paletas 2009; Fabbrini et al. 2010).

In addition to environmental risk factors, NAFLD may have genetic risk factors. Specifically, there appears to be differences in risk depending on ethnic background. Based on recent population‐based studies in the United States, the prevalence of NAFLD was 30–32% overall, with rates of 39–45% among Hispanics, 30–33% among non‐Hispanic Whites, and 23–24% among non‐Hispanic Blacks (Browning et al. 2004; Smits et al. 2013). Multiple studies have indeed illustrated the lower disease burden and decreased histologic severity in the African‐American population (Giday et al. 2006; Kallwitz et al. 2009; Mohanty et al. 2009). Interestingly, although individuals of Asian ancestry tend to have lower rates of obesity, they have high rates of metabolic syndrome and NAFLD (prevalence ranging from 15 to 45%) (Farrell et al. 2013; Wong and Ahmed 2014). Asians may even demonstrate a higher disease severity than whites (Mohanty et al. 2009). A number of studies have demonstrated that Hispanics in the United States have both a higher disease burden and an increased disease severity than their counterparts of African‐American and European ancestry (Clark et al. 2003; Neuschwander‐Tetri and Caldwell 2003; Weston et al. 2005; Kallwitz et al. 2009; Sharp et al. 2009; Wagenknecht et al. 2009; Pan et al. 2011; Schneider et al. 2013). These studies investigated primarily Mexican Americans, but not Hispanics from other locations (Browning et al. 2004; Mohanty et al. 2009). This presents a complication when attempting to apply genetic and clinical principles derived from research in different Hispanic subpopulations. In the Bronx, for instance, 53.5% of the county is of Hispanic ethnicity, but over 80% are of Caribbean (Dominican and Puerto Rican) ancestry (2008–2012 American Community Survey). One study recently acknowledged the shortcoming of these NAFLD‐Hispanic investigations and analyzed rates of “suspected” NAFLD among different Hispanic populations. They discovered that rates of the condition differed between ancestries, with Hispanics of Cuban, Puerto Rican, and Dominican backgrounds with lower prevalence of NAFLD when compared with those of Mexican heritage (Kallwitz et al. 2015).

Here, we performed a new study to investigate the potential genetic risk implicated in NAFLD within a population composed of Hispanics of mostly Caribbean ancestry in Bronx County. We compared the allele frequencies of genetic variations in candidate genes found for NAFLD to those in our local population.

## Materials and Methods

### Human subjects and phenotype data

Blood or saliva samples were obtained from 40 patients with biopsy‐proven NAFLD, with their informed consent (IRB # 11‐06‐247E). The 12 males and 28 females are all of self‐identified Hispanic descent and are over the age of 18. Control samples (*n *=* *24) were obtained from patients visiting various primary care, dermatology, and gastroenterology clinics at Montefiore Medical Center in the Bronx, New York. Subjects included 12 females and 12 males self‐identified as Hispanic. Patients were excluded if they had any evidence of liver disease (abnormal abdominal imaging, AST/ALT levels, or clinical history), BMI >30, or other evidence of metabolic syndrome. If patients had isolated HTN, hypercholesterolemia, or controlled DM, they were included based on clinical judgment.

Blood samples were obtained from healthy parents of children seen at the Pediatric Genetics Clinic at Montefiore Medical Center for developmental delay, autism, or multiple congenital malformations (mostly sporadic). Their DNA was used as a proxy for a random sample of our Bronx population. Their ethnic makeup was selected to be reflective of that of Bronx County, NY:−43% Hispanic, 31% White, 15.9% Black, 5% Asian. Subjects were not screened for NAFLD or other exclusion criteria. Deidentified samples were also obtained with their informed consent (IRB #1999‐201).

The Gentra Puregene Genomic DNA Purification Kit (Gentra, Minneapolis, MN) was used to purify DNA in the Molecular Cytogenetics Core, Albert Einstein College of Medicine, NY, according to standard protocols. Specimens were derived from blood (purple top EDTA tubes) or saliva (collected in Oragene OR‐250 kits). Quality of DNA was visualized by agarose gel electrophoresis and quantified by nanodrop analysis, Qubit, and/or PicoGreen.

### Genotype analysis

Based on prior GWAS and candidate gene studies, we compiled a list of 87 target SNPs in 52 genes associated with the development NAFLD or disease severity. Seventy‐four SNPs (Table 1) were included in the study design. The other SNPs were rejected if there was a nearby SNP within 20 bases of the target SNP or the nearby SNP blocked the design of an extension primer. We subsequently performed SNP‐based genotyping with SNPs using Sequenom MassArray technology.

**Table 1:** (A) GWAS – target SNPs (25). (B) Candidate gene and association studies (52). (C): SNPs excluded from study design (13)

SNP	HGVS name	Gene	Risk allele	*N*	*P*‐value
(A)					
rs2896019 (Kawaguchi et al. 2012)	NM_025225.2:c.979+542T>G	*PNPLA3*	G	543	7.5E‐10
rs738409 (Chambers 2011; Speliotes 2011; Kawaguchi et al. 2012)	NM_025225.2:c.444C>G	*PNPLA3*	G	543–61089	(1E‐10) – (1E‐45)
rs6006460 (Romeo et al. 2008)	NM_025225.2:c.1358G>T	*PNPLA3*	G	9229	0.0007
rs11597390 (Yuan et al. 2008)	NC_000010.11:g.100101678G>A	*Intergenic*	A	7715	2.9E‐08
rs1227756 (Chalasani et al. 2010)	NM_080805.3:c.364+6310G>A	*COL13A1*	G	236	2E‐07
rs10883437 (Chambers 2011)	NC_000010.11:g.100035604T>G	*Intergenic*	T	61089	4E‐09
rs2710833 (Chalasani et al. 2010)	NC_000010.11:g.100035604T>G	*DDX60L*	A	236	6.3E‐07
rs887304 (Chalasani et al. 2010)	NM_001144958.1:c.1118+160A>G	*CRACR2A*	A	236	7.7E‐07
rs6591182 (Chalasani et al. 2010)	NM_001099409.1:c.1613T>G	*EHBP1L1*	A	236	8.6E‐07
rs780094 (Kawaguchi et al. 2012)	NM_001486.3:c.1423‐418T>C	*GCKR*	T	543	0.011
rs343062 (Chalasani et al.^2010^)	NC_000007.14:g.35509456C>T	*HERPUD2*	T	236	2.7E‐08
rs6834314 (Chambers 2011)	NC_000004.12:g.87292656A>G	*HSD17B13*	A	61089	3.1E‐09
rs12137855 (Speliotes 2011)	NC_000001.11:g.219275036C>T	*LYPLAL1*	C	592	4.12E‐05
rs2228603 (Speliotes 2011)	NM_004386.2:c.274C>T	*NCAN*	T	592	5.29E‐05
rs6006611 (Kitamoto et al. 2013)	NM_001003828.2:c.211+5145G>A	*PARVB*	G	392	1.8E‐12
rs343064 (Chalasani et al. 2010)	NC_000007.14:g.35515178C>T	*PDGFA*	A	236	3E‐08
rs4240624 (Speliotes 2011)	NR_040039.1:n.765+418G>A	*PPP1R3B*	A	592	3.68E‐18
rs6487679 (Chalasani et al. 2010)	NC_000012.12:g.9218736C>T	*PZP*	G	236	0.000001
rs2143571 (Kawaguchi et al. 2012)	NM_015380.4:c.1365‐532G>A	*SAMM50*	A	543	6.4E‐07
rs738491 (Kawaguchi et al. 2012)	NM_015380.4:c.21+2633C>T	*SAMM50*	T	543	3.9E‐06
rs3761472 (Kawaguchi et al. 2012)	NM_015380.4:c.329A>G	*SAMM50*	G	543	1.1E‐06
rs2954021 (Chambers 2011)	NC_000008.11:g.125469835A>G	*TRIB1*	A	61089	2.3E‐13
rs2645424 (Chalasani et al. 2010)	NM_001287756.1:c.201+739A>G	*FDFT1*	A	236	6.8E‐07
rs2499604 (Chalasani et al. 2010)	NC_000001.11:g.237940201C>T	*ZP4*	A	236	0.000002
rs343062 (Chalasani et al. 2010)	NC_000007.14:g.35509456C>T	*HERPUD2*	T	236	2.70E‐08
(B)					
rs8187710 (Sookoian et al. 2009)	NM_000392.4:c.4544G>A	*ABCC2*	G	109	0.035
rs17222723 (Sookoian et al. 2009)	NM_000392.4:c.3563T>A	*ABCC2*	A	109	0.037
rs2241766 (Tokushige et al. 2009)	NM_004797.3:c.45T>G	*ADIPOQ*	T	70	0.0002
rs1501299 (Musso et al. 2008)	NM_004797.3:c.214+62G>T	*ADIPOQ*	G	119	0.03
rs767870 (Kotronen et al. 2009)	NM_024551.2:c.650+20G>A	*ADIPOR2*	T	302	0.024
rs3772627 (Yoneda et al. 2009)	NM_031850.3:c.‐1+4440A>G	*AGTR1*	C	167	4.1E‐06
rs3772633 (Yoneda et al. 2009)	NM_031850.3:c.‐85+2254T>C	*AGTR1*	G	167	6.5E‐06
rs3772630 (Yoneda et al. 2009)	NM_031850.3:c.‐1+658T>C	*AGTR1*	G	167	0.000042
rs2276736 (Yoneda et al. 2009)	NM_031850.3:c.‐1+59A>G	*AGTR1*	C	167	0.000047
rs2854116 (Petersen et al. 2010)	NM_000040.1:c.‐501C>T	*APOC3*	C	95	0.001
rs2854117 (Petersen et al. 2010)	NM_000040.1:c.‐528T>C	*APOC3*	T	163	0.02
rs3772622 (Yoneda et al. 2009; Zain et al. 2013b)	NM_031850.3:c.‐1+9939T>C	*ATGR1*	C	144–167	0.003–1.2E‐06
rs11591741 (Feitosa et al. 2013)	NM_001278.3:c.933+1251C>G	*CHUK*	C	2705	3.43E‐06
rs11597086 (Feitosa et al. 2013)	NM_001278.3:c.1974+36T>G	*CHUK*	C	2705	3.07E‐05
rs6850524 (Sookoian et al. 2007)	NM_004898.3:c.‐289‐5765G>C	*CLOCK*	G	136	0.00899
rs6843722 (Sookoian et al. 2007)	NM_152760.2:c.1073‐31G>A	*CLOCK*	C	136	0.0229
rs4864548 (Sookoian et al. 2007)	NM_004898.3:c.‐1144C>T	*CLOCK*	A	136	0.02697
rs11932595 (Sookoian et al. 2007)	NM_004898.3:c.793‐1130T>C	*CLOCK*	A	136	0.0449
rs28969387 (Vazquez‐Chantada et al. 2013)	NM_000773.3:c.1370A>T	*CYP2E1*	A	69	1.56E‐07
rs1044498 (Dongiovanni et al. 2010)	NM_006208.2:c.517A>C	*ENPP1*	C	702	<0.05
rs2862954 (Feitosa et al. 2013)	NM_006459.3:c.871A>G	*ERLIN1*	C	2705	5.74E‐06
rs1260326 (Tan et al. 2013)	NM_001486.3:c.1337T>C	*GCKR*	T	144	0.012
rs17883901 (Oliveira et al. 2010)	NM_001498.3:c.‐594C>T	*GCLC*	T	130	0.007
rs12979860 (Petta et al. 2012)	NM_001276254.2:c.151‐152G>A	*IL28B*	C	74	<0.001
rs1800795 (Carulli et al. 2009)	NM_000600.3:c.‐237C>G	*IL6*	C	114	<0.01
rs1801278 (Dongiovanni et al. 2010)	NM_005544.2:c.2911G>A	*IRS1*	C	702	<0.05
rs7324845 (Adams et al. 2013)	NM_002298.4:c.1752‐1284C>T	*LCP1*	A	13	2.96E‐06
rs6700896 (Swellam and Hamdy 2012)	NM_002303.5:c.2673+1118C>T	*LEPR*	T	90	0.0001
rs1137100 (Zain et al. 2013a)	NM_002303.5:c.326A>G	*LEPR*	G	144	0.003
rs1137101 (Zain et al. 2013a)	NM_002303.5:c.668A>G	*LEPR*	G	144	0.013
rs13412852 (Valenti et al. 2012)	NM_145693.2:c.722+1070C>T	*LPIN1*	C	142	<0.01
rs12743824 (Adams et al. 2013)	NC_000001.11:g.99317401C>A	*LPPR4*	C	13	4.82E‐06
rs1800591 (Namikawa et al. 2004)	NM_001300785.1:c.143‐7574G>T	*MTTP*	G	63	0.002
rs3816873 (Hashemi et al. 2011)	NM_001300785.1:c.464T>C	*MTTP*	C	83	0.05
rs1800804 (Peng et al. 2013)	NM_001300785.1:c.143‐7245T>C	*MTTP*	C	580	0.05
rs694539 (Sazci et al. 2013)	NC_000011.9:g.114133419C>T	*NNMT*	A	80	0.051
rs2461823 (Sookoian et al. 2010)	NM_033013.2:c.‐22‐5951T>C	*NR1I2*	A	188	0.039
rs7946 (Song et al. 2005; Dong et al. 2007)	NM_148173.1:c.523G>A	*PEMT*	A	28–107	<0.01–0.03
rs738409 (Romeo et al. 2008)	NM_025225.2:c.444C>G	*PNPLA3*	G	2111	5.9E‐10
rs2126259 (Feitosa et al. 2013)	NR_040039.1:n.765+1333T>C	*PPP1R3B*	G	2705	4.81E‐08
rs1800234 (Chen et al. 2008)	NM_005036.4:c.680T>C	*PPARA*	T	79	0.011
rs8192678 (Lin et al. 2013)	NM_013261.3:c.1444G>A	*PPARGC1A*	A	182	0.008
rs7643645 (Sookoian et al. 2010)	NM_033013.2:c.‐22‐579A>G	*PXR*	G	188	<0.0015
rs56225452 (Auinger et al. 2010)	NM_012254.2:c.‐1324G>A	*SLC27A5*	A	103	0.02
rs2229682 (Vazquez‐Chantada et al. 2013)	NM_006516.2:c.588G>C	*SLC2A1*	A	69	0.000912
rs11864146 (Adams et al. 2013)	NM_001080442.2:c.1163‐58T>C	*SLC38A8*	A	13	1.86E‐06
rs4880 (Al‐Serri et al. 2012)	NM_001024466.1:c.47T>C	*SOD2*	T	55	0.038
rs6503695 (Sookoian et al. 2008)	NM_213662.1:c.129‐802A>G	*STAT3*	C	108	0.021
rs780094 (Yang et al. 2011; Tan et al. 2013)	NM_001486.3:c.1423‐418T>C	*GCKR*	T	144–436	0.013–0.0072
rs7903146 (Musso et al. 2009)	NM_030756.4:c.382‐41435C>T	*TCF7L2*	T	39	0.0001
rs11235972 (Xu et al. 2013)	NM_022803.2:c.338‐96C>T	*UCP3*	G	250	0.03
rs1800849 (Aller et al. 2010)	NM_022803.2:c.‐238C>T	*UCP3*	T	39	<0.05
(C)					
rs12447924 Adams et al. 2012);	NM_001286085.1:c.‐1700C>T	*CETP*	T	467	3.0E‐04
rs12597002 (Adams et al. 2012)	NM_001286085.1:c.234‐894C>A	*CETP*	A	467	2.0E‐04
rs1800471 (Dixon et al. 2003)	NM_000660.5:c.74G>C	*TGF‐β1*	G	105	0.77
rs57920071 (Ludtke et al. 2005)	NM_170708.3:c.1444C>T	*LMNA*	T	18	Not Given
rs3750861 (Miele et al. 2008)	NM_001300.5:c.103‐27G>A	*KLF6*	A	415	4.0E‐03
rs1554483 (Sookoian et al. 2007)	NM_004898.3:c.982+247G>C	*CLOCK*	G	136	0.02697
rs9891119 (Sookoian et al. 2008)	NM_213662.1:c.‐23‐7423T>G	*STAT3*	C	108	0.02
rs1799964 (Tokushige et al. 2007)	NM_000594.3:c.‐1211T>C	*TNF*	C	102	<0.01
rs1800630 (Tokushige et al. 2007)	NM_000594.3:c.‐1043C>A	*TNF*	A	102	<0.01
rs1799945 (Valenti et al. 2010a,b)	NM_139011.2:c.77‐2168C>G	*HFE*	G	587	<0.005
rs1800562 (Valenti et al. 2010a,b)	NM_139011.2:c.77‐206G>A	*HFE*	A	587	<0.005
rs361525 (Valenti et al. 2002)	NM_000594.3:c.‐418G>A	*TNF*	A	99	<0.0001
rs2290602 (Yoneda et al. 2008)	NM_013261.3:c.877+171A>C	*PPARGC1A*	T	115	<0.02

GWAS, genome‐wide association study; SNP, single‐nucleotide polymorphisms.

Additionally, we sequenced the *PNPLA3* (OMIM # 609567) gene including SNP rs738409 (NM_025225.2:c.444C>G) in exon 3, which is the most consistently and significantly associated variation with the development of NAFLD (Anstee and Day 2013). To detect sequence variations, we amplified the nine coding exons of *PNPLA3* using PCR followed by Sanger DNA sequencing. Primers were designed specifically for each exon, based on GenBank reference sequence (Table S1). PCR amplified products were purified using the AmpPure Purification System processed by Beckman Coulter BioMan and carried out according to the manufacturer's protocol (Beckman Coulter, Indianapolis, IN). Purified PCR products were sequenced on the Applied Biosystems 3730 Sequencer (Genomics Core, Einstein, NY). All sequence data were compiled using Sequencher 4.0.1 software and compared to reference sequence NM_025225.2 (Gene Codes, Ann Arbor, MI).

### Statistical analysis

Allele frequencies of the variations were estimated by the gene‐counting method. Agreement with the Hardy–Weinberg equilibrium was tested by the chi‐square test. Allele frequencies between each group were also compared using the chi‐square test. PLINK software was used to evaluate all Sequenom results. All other analyses were performed in Microsoft Excel.

## Results

### Target variation analysis

Sequenom genotyping and *PNPLA3* sequencing were used to identify allele frequencies of the target SNPs within NAFLD cases, controls, and our sample Bronx population. Additional minor allele frequencies (MAFs) were determined through 1000 Genomes Project Phase 1 report of the total population as well as subpopulation of Puerto Ricans from Puerto Rico. Complete results are included in Table S2. Nine variations are listed in Table 2 based on significant *P*‐values (≤0.05) across multiple avenues of comparison.

**Table 2:** Selected genotyping results. (A) Comparisons relative to NAFLD cases. (B) comparisons relative to sample Bronx population

(A)														
Gene	SNP	MAF‐NAFLD cases	MAF‐controls	OR	*P*‐value	MAF‐Bronx population	OR	*P*‐value	MAF‐general population	RR	*P*‐value	MAF‐Puerto Rican population	OR	*P*‐value
CHUK	rs11597086	26% (22/15/3)	16% (15/7/0)	1.65	0.35	23% (134/75/14)	1.14	0.67	22% (694/308/90)	1.18	0.55	43% (19/25/11)	0.61	0.09
ENPP1	rs1044498	37% (19/11/9)	42% (10/8/6)	0.89	0.72	36% (105/80/42)	1.03	0.90	29% (660/224/208)	1.27	0.29	19% (37/15/3)	1.96	0.05
ERLIN1	rs2862954	29% (20/14/4)	14% (15/6/0)	2.03	0.20	26% (127/69/22)	1.12	0.70	25% (665/314/113)	1.17	0.55	48% (15/27/13)	0.60	0.07
PNPLA3	rs738409	56% (9/17/14)	19% (16/7/1)	2.95	0.003	19% (139/62/8)	3.01	4.4E‐7	28% (590/382/120)	1.97	0.0001	36% (20/30/5)	1.56	0.05
PNPLA3	rs2896019	41% (17/13/10)	17% (17/6/1)	2.48	0.04	25% (132/75/20)	1.63	0.04	28% (604/374/114)	1.50	0.06	28% (27/25/3)	1.47	0.18
SAMM50	rs3761472	38% (17/14/8)	17% (16/8/0)	2.31	0.07	21% (146/68/14)	1.83	0.02	25% (628/374/90)	1.52	0.07	21% (33/21/1)	1.83	0.06
SAMM50	rs2143571	44% (14/16/9)	25% (13/10/1)	1.74	0.14	28% (120/88/20)	1.55	0.05	32% (514/453/125)	1.35	0.14	30% (25/27/3)	1.45	0.18
ABCC2	rs17222723	10% (31/8/0)	2% (23/1/0)	4.92	0.22	7% (193/33/0)	1.40	0.52	4% (1000/91/1)	2.40	0.08	7% (47/8/0)	1.47	0.57

(B)								
		MAF‐Bronx population	MAF‐general population	OR	*P*‐value	MAF‐Puerto Rican population	OR	*P*‐value
CHUK	rs11597086	23%	22%	1.04	0.79	43%	0.54	0.003
ENPP1	rs1044498	36%	29%	1.24	0.04	19%	1.90	0.02
ERLIN1	rs2862954	26%	25%	1.05	0.69	48%	0.54	1.5E‐3
PNPLA3	rs738409	19%	28%	0.66	3.5E‐3	36%	0.52	5.2E‐4
PNPLA3	rs2896019	25%	28%	0.92	0.49	28%	0.90	0.68
SAMM50	rs3761472	21%	25%	0.83	0.17	21%	1.00	0.98
SAMM50	rs2143571	28%	32%	0.87	0.22	30%	0.94	0.78
ABCC2	rs17222723	7%	4%	1.71	0.05	7%	1.04	0.94

NAFLD, nonalcoholic fatty liver disease; SNP, single‐nucleotide polymorphisms.

The SNPs in *PNPLA3* and *SAMM50* (OMIM # 612058) (rs738409, rs2896019; NM_025225.2:c.979+542T>G, rs3761472; NM_015380.4:c.329A>G) were previously identified in GWAS studies and generally had increased frequencies in the NAFLD population compared with our controls, the sample Bronx population, and expected population frequencies. Increased MAF of the alternative allele of SNPs rs738409 and rs2896019 in *PNPLA3* was found in our Bronx population sample as compared to the MAF recorded for the Puerto Rican population. Similarly, the MAF of the alternative allele was increased significantly in disease‐related SNPs, rs1044498 ((NM_006208.2:c.517A>C) and rs17222723 (NM_000392.4:c.3563T>A), in *ENPP1* (OMIM # 173335) and *ABCC2* (OMIM # 601107). The MAF of the alternative allele of rs17222723 and rs1044498 were statistically significant (*P* < 0.05) in both the NAFLD cases and Bronx population than the expected based on population data, but were not significant when comparing NAFLD cases versus Bronx population. The SNP rs1044498 (*ENPP1)* was most significant (*P* = 0.02) when comparing the Bronx population with the Puerto Rican population.

Common SNP variations in an intron of *CHUK* (OMIM # 600664) (rs11597086; (NM_001278.3:c.1974+36T>G)) and exon of *ERLIN1* (OMIM # 611604) (rs2862954; (NM_001100626.1:c.871A>G)) occurred less frequently in both the NAFLD cases and the sample Bronx population than would be expected in a random Puerto Rican population. The intronic *CHUK*, SNP variant, rs11591741, also occurred less frequently in our Bronx population than would be expected in a random Puerto Rican population (31 vs. 43%, *P* = 0.03); however, it occurred more often than would be expected in the general population (31 vs. 23%, *P* = 0.004).

### PNPLA3 sequencing

Sixteen common and six rare DNA variations were uncovered upon sequencing of *PNPLA3* (Table 3). Two SNPs, rs738409 and rs6006460 (NM_025225.2:c.1358G>T), have known associations with NAFLD genes from previous GWAS studies and were also included in our Sequenom assay; three other SNPs are associated with hepatic steatosis as suggested in the literature (rs738408; NM_025225.2:c.447C>T (Speliotes 2011), rs4823173; NM_025225.2:c.487‐28G>A (DiStefano et al. 2014), rs139051; NM_025225.2:c.421‐28A>G (Peng et al. 2012)). The remaining 17 SNP variations have no known disease or phenotypic associations based on literature review or Online Mendelian Inheritance in Man (OMIM). Six variants with a MAF of ≤1% in the general, Hispanic, and Puerto Rican populations were discovered and included in Table 3. Of note, four of these variations occurred more often in our NAFLD cases than in controls (rs144730517; NM_025225.2:c.1184C>G, rs192841252; NM_025225.2:c.420+23C>T, rs41278871; NM_025225.2:c.*393G>C, rs144821153 NM_025225.2:c.‐40C>G).

**Table 3:** PNPLA3 sequencing results (all *P*‐values are for relative comparisons to NAFLD cases)

SNP	NAFLD allele frequency *n* = 40	Control allele frequency *n* = 24	*P*	OR	Minor allele	Chromosome position	MAF *n* = 2184	*P*	MAF (HIS) *n* = 362	*P*	MAF (PUR) *n* = 110	*P*
rs738409	56%	19%	0.003	3.00	G	43,928,847	28.4%	1.18E‐04	45%	0.18	36%	0.03
rs738408	55%	19%	0.004	2.93	T	43,928,850	28.4%	2.35E‐04	45%	0.23	36%	0.04
rs2294919	10%	30%	0.040	0.33	T	43,946,445	26.1%	0.02	19%	0.16	19%	0.19
rs4823173	41%	19%	0.063	2.20	A	43,932,850	25.7%	0.03	39%	0.78	28%	0.12
rs139047	35%	14%	0.086	2.45	A	43,927,194	34.2%	0.92	25%	0.17	25%	0.23
rs2294918	20%	39%	0.112	0.52	A	43,946,236	23.0%	0.65	24%	0.57	31%	0.18
rs139051	54%	38%	0.208	1.43	A	43,928,796	47.8%	0.46	55%	0.88	45%	0.34
rs34879941	25%	8%	0.215	3.00	T	43,936,998	27.3%	0.75	40%	0.06	28%	0.72
rs41278873	10%	2%	0.246	4.60	C	43,946,811	3.0%	0.01	6%	0.33	9%	0.85
rs144730517	3%	0%	0.353	2.56^a^	G	43,944,762	0.1%	6.94E‐05	0%	0.00	0%	0.10
rs115043594	0%	2%	0.445	0.29	A	43,946,514	0.0%	NA	*NA*	*NA*	*NA*	*NA*
rs192841252	4%	0%	0.614	2.15	T	43,927,190	0.1%	1.85E‐08	1%	0.14	1%	0.25
rs2294917	16%	21%	0.652	0.77	C	43,946,106	35.2%	0.01	28%	0.11	29%	0.11
rs2076213	14%	10%	0.696	1.32	G	43,927,042	7.6%	0.15	17%	0.60	13%	0.90
rs41278871	3%	0%	0.743	1.68	C	43,946,775	0.6%	0.13	0%	0.00	0%	0.10
rs2072906	23%	18%	0.758	1.24	G	43,937,292	27.5%	0.48	40%	0.03	28%	0.50
rs2076212	14%	17%	0.760	0.83	T	43,927,090	11.0%	0.58	13%	0.89	15%	0.87
rs6006461	1%	0%	0.793	1.56	T	43,946,433	0.2%	0.78	0%	*NA*	0%	*NA*
rs76510336	3%	2%	0.797	1.55	G	43,946,295	1.4%	0.56	0%	0.00	1%	0.49
rs6006460	1%	2%	0.898	0.78	T	43,946,294	3.1%	0.26	0%	*NA*	0%	*NA*
rs144821153	1%	0%	0.908	1.22	G	43,923,872	0.4%	0.41	1%	0.88	1%	0.85
rs34179073	3%	2%	0.915	1.20	T	43,932,952	4.4%	0.56	7%	0.28	6%	0.39

NAFLD, nonalcoholic fatty liver disease; MAF, minor allele frequency; SNP, single‐nucleotide polymorphisms.

a Using zero‐cell correction.

## Discussion

NAFLD is a highly prevalent disease that is the most common cause of liver disease worldwide. With its connection to obesity and metabolic syndrome, NAFLD and its more severe form, NASH, are projected to continually increase as a public health burden. Although NAFLD occurs with increased incidence and severity in the Mexican Hispanic population, the principal aim of this study was to explore previously discovered NAFLD variants in our largely Caribbean–Hispanic population. The NAFLD patients seen at Montefiore Medical Center are of mostly Dominican or Puerto Rican ancestry, populations highly affected with fatty liver disease and to our knowledge never studied in this context. We performed liver biopsies as part of the clinical evaluation of these patients. Additionally, our analysis benefits from the inclusion of previously discovered and/or validated genes and variants related to fatty liver disease by providing more power. This may enhance any implications made in the context of our limited number of samples. Indeed, the greatest weakness in our study is the relatively small number of patients when examining a common disease. Therefore, the purpose of our discussion is to provide a framework for future analyses. All results of our experiments are suggestive, but not intended to be conclusive.

As discussed previously, NAFLD is a complex disease with a multifactorial etiology associated with diet, obesity, and a variety of comorbidities related to insulin resistance (Marchesini et al. 1999). Although environmental factors are important in determining risk for the disease, evidence from familial and twin studies support the assumption that genetics provide an important modulator of NAFLD development and disease progression (Brouwers et al. 2006; Makkonen et al. 2009; Schwimmer et al. 2009). The most studied and replicated variant is rs738409 (NM_025225.2:c.444C>G) in *PNPLA3*. This common missense variant has demonstrated increased risk for the development of NAFLD independent of diabetes or obesity and is also significantly associated with degree of histological severity (Rotman et al. 2010; Speliotes et al. 2010; Valenti et al. 2010a,b). The strongest effect of the variant appears to exist within the Hispanic population versus African‐American or European‐American individuals (Romeo et al. 2008). The overall strength of the association between this polymorphism and NAFLD has recently been confirmed in a genetic meta‐analysis (Sookoian and Pirola 2011).

Although the missense SNP, rs738409, has been repeatedly evaluated and tested, to our knowledge the entire coding region of *PNPLA3* had never been sequenced in the Hispanic population with respect to NAFLD. Our study demonstrated that the minor, G allele is more common in our Caribbean–Hispanic patients with NAFLD than in any other group analyzed. This included ethnically matched controls (OR 2.95, *P* = 0.003), the sample Bronx population (OR 3.01, *P* = 4.4E‐7), the general population (OR 1.97, *P* = 0.0001), and even the average Puerto Rican population (OR 1.56, *P* = 0.05).

Mutations in *PNPLA3* provide a possible genetic basis for the underlying mechanisms in the genesis of NAFLD. The *PNPLA3* gene is located on chromosome 22q13.3 and encodes for a membrane‐bound protein mediating triacylglycerol hydrolysis in adipocytes. Its expression is highest in human liver tissue (Huang et al. 2010) and is induced during feeding and insulin resistance by fatty acids and other regulators of lipogenesis (Huang et al. 2010; Dongiovanni et al. 2013). The rs738409 missense variation specifically is assumed to promote triglyceride accumulation through relative inhibition of triglyceride hydrolysis (He et al. 2010). An understanding of the theoretical pathophysiology behind NAFLD illustrates how *PNPLA3*'s function is consistent with a theoretical role in the progression of the disease.

Although the exact pathogenesis of NAFLD is still under debate, there are a number of mechanisms that are clearly involved. These include insulin resistance, free fatty acid flux, endoplasmic reticulum stress, oxidative stress, and inflammation (Yoon and Cha 2014). Simplistically, steatosis in the liver develops when supply of fatty acids to the liver exceed the demand in requirements for mitochondrial oxidation and synthesis of phospholipids, triglycerides, and cholesterol (Lall et al. 2008). Triglyceride accumulation results from either lipid uptake in the liver or de novo synthesis in the setting of excess carbohydrates (Kawano and Cohen 2013). Insulin resistance has repeatedly been implicated as an important cause of lipid accumulation in the liver (Marchesini et al. 1999; Sanyal et al. 2001; Chitturi et al. 2002; Pagano et al. 2002). Once the liver is overrun with lipid accumulation, the mitochondria attempt to remove the fatty acids through oxidation. However, this process can inadvertently cause oxidative stress and mitochondrial dysfunction through excessive production of reactive oxygen species (ROS) (Rolo et al. 2012; Yoon and Cha 2014). This hepatocellular injury is further exacerbated by the secretion of inflammatory cytokines (e.g., TNF‐*α*, IL‐6, and NO) that are induced through the presence of adipose tissue in the liver and basal insulin resistance (Carter‐Kent et al. 2008; Hijona et al. 2010; Odegaard and Chawla 2011; Yoon and Cha 2014). This proinflammatory state and resultant fibrosis/necrosis define the histopathological spectrum of NAFLD.

Many of the genes identified in our study as having variations with multiple significant results have functions that fit in with the pathogenesis of NAFLD and may logically influence disease susceptibility or progression. These particularly include *SAMM50*,* ENPP1,* and *ABCC2*. *ENPP1*'s function can be directly linked to hepatic steatosis as it codes for a membrane glycoprotein functioning to inhibit insulin signaling. The rs1044498, SNP variation causes a gain‐of‐function mutation that causes overexpression in peripheral insulin target tissues and is associated with human insulin resistance (Prudente and Trischitta 2006). If *SAMM50* is involved in NAFLD, it is likely related to its role in mitochondrial function. *SAMM50* encodes a component of the SAM assembly complex, which helps integrate *β*‐barrel proteins into the outer mitochondrial membrane. Any mitochondrial dysfunction and resultant decrease in removal of reactive oxygen species (ROS) as a result of mutations in *SAMM50* is consistent with biochemical rationale for the importance of *SAMM50* in NAFLD (Kitamoto et al. 2013). *ABCC2* has a function that is more removed from a direct correlation with NAFLD. It encodes a protein expressed in the apical area of the hepatocyte and functions in biliary transport, likely critical to the elimination of conjugates of many toxins from hepatocytes into bile (Nies and Keppler 2007) and likely predisposing liver toward injury from excessive adipose tissue (Sookoian et al. 2009).

There is one gene cluster, *ERLIN1‐CHUK‐CWF19L1*, which revealed associations with SNPs that appear to confer protection in our study. The relation of these genes to NAFLD is theoretically logical as *ERLIN1* comprises a component of lipid rafts (Browman et al. 2006) and *CHUK* proteins modulate NF‐kB activation of several genes involved in insulin resistance (Yuan et al. 2008). For the SNPs rs11597086 and rs2862954, the rate of polymorphism in our sample Bronx population was lower than in the average Puerto Rican population (OR 0.54, *P* = 0.003; OR 0.54, *P* = 1.5E‐3). In our study, these variations also occur less frequently in NAFLD cases than would be expected in a random Puerto Rican population (OR 0.61, *P* = 0.09, OR 0.60, *P* = 0.07). The fact that these polymorphisms only seem to confer protection when compared with the Puerto Rican community suggest that there is some other genetic modifier in the Hispanic subpopulation that interacts with these genes to protect patients from the development of hepatic steatosis. This phenomenon is interesting and warrants further investigation.

In conclusion, this study implies significant interactions of variants from *PNPLA3*,* ERLIN1*‐ *CHUK*,* SAMM50*,* ENPP1*, and *ABCC2* with NAFLD in Hispanics from a majority Caribbean ancestry. An interaction of these genes with NAFLD is pathophysiologically plausible and the extent of impact of these variations in NAFLD generally and in sub‐Hispanic populations deserves further analysis.

## Conflict of Interest

None declared.

## Supporting information


**Table S1**. PNPLA3 primers.
**Table S2**. Full sequenom results (Target SNPs).
