# Supplementary material for: Genetic analysis of nonalcoholic fatty liver disease within a Caribbean–Hispanic population
Source: Mol Genet Genomic Med. 2015 Aug 11;3(6):558–69. doi: 10.1002/mgg3.168 (PMC4694126; doi:10.1002/mgg3.168)
Supplement: Supplementary file 1 — Table S1. PNPLA3 primers. Table S2. Full sequenom results (Target SNPs). [file MGG3-3-558-s001.docx]

**Supplemental Info**

Supplementary Table I - PNPLA3 Primers

|  | Forward Primer | Reverse Primer |
| --- | --- | --- |
| Exon 1 | 5' ttggaaaaatgctttctctcg 3' | 5' cacgagagtcccaggcttc 3' |
| Exon 2 | 5' tctggccttgccaaaagtat 3' | 5' cacacccatcccaaaaca 3' |
| Exon 3 | 5' ccctgctcacttggagaaag 3' | 5' tgtgtgagcacacttcagagg 3' |
| Exon 4 | 5' ctgagaagctctgcccacat 3' | 5' caatcagggaggcaaagaag 3' |
| Exon 5 | 5' tgcatgattctttggtgctc 3' | 5' ccaagaccgctagtaaatgctt 3' |
| Exon 6 | 5' ccccttgcatttggctaata 3' | 5' ggcagtaccatcttcctcca 3' |
| Exon 7 | 5' ggaaatgctaagcaccaaca 3' | 5' cccagactacatgccaccat 3' |
| Exon 8 | 5' aggtgatgttggcagctttt 3' | 5' ggtgttcagccagctgtttc 3' |
| Exon 9-3UTR1 | 5' caatgtgggtccaccgtag 3' | 5' CAGGGGTCACTACACAGCAA 3' |
| 3UTR2 | 5' GAGGGGCTCTCCACCTTTC 3' | 5' TCAACAGGTAACAACGCTTCC 3' |
| 3UTR3 | 5' GCGGGGGTAACAAGATGATA 3' | 5' acattcagggccactaccg 3' |

Supplementary Table II - Full Sequenom Results (Target SNPs)

| Gene | SNP | MAF-  *NAFLD Cases* | MAF-  *Controls* | MAF-  *Bronx Population* | MAF-  *General Population* | MAF-  *Puerto Rican Population* |
| --- | --- | --- | --- | --- | --- | --- |
| ABCC2 | rs17222723 | 10% | 2% | 7% | 4% | 7% |
| ABCC2 | rs8187710 | 89% | 93% | 88% | 93% | 89% |
| ADIPOQ | rs1501299 | 75% | 68% | 72% | 68% | 70% |
| ADIPOQ | rs2241766 | 78% | 80% | 86% | 86% | 81% |
| ADIPOR2 | rs767870 | 79% | 48% | 73% | 75% | 83% |
| AGTR1 | rs3772630 | 48% | 48% | 49% | 43% | 35% |
| AGTR1 | rs3772633 | 20% | 14% | 17% | 17% | 17% |
| AGTR1 | rs2276736 | 44% | 42% | 41% | 38% | 34% |
| AGTR1 | rs3772627 | 48% | 50% | 46% | 43% | 35% |
| ALTQTL2 | rs11597390 | N/A | N/A | N/A | 25% | 41% |
| APOC3 | rs2854116 | 44% | 59% | 52% | 49% | 40% |
| APOC3 | rs2854117 | 36% | 54% | 41% | 46% | 35% |
| ATGR1 | rs3772622 | 41% | 33% | 31% | 37% | 46% |
| CD14 | rs2569190 | N/A | N/A | 54% | 47% | 50% |
| CHUK | rs11597086 | 26% | 16% | 23% | 22% | 43% |
| CHUK | rs11591741 | N/A | N/A | 31% | 23% | 43% |
| CLOCK | rs11932595 | 31% | 50% | 39% | 33% | 57% |
| CLOCK | rs4864548 | 30% | 30% | 36% | 39% | 38% |
| CLOCK | rs6843722 | 30% | 31% | 35% | 36% | 36% |
| CLOCK | rs6850524 | 63% | 76% | 57% | 57% | 70% |
| COL13A1 | rs1227756 | 57% | 60% | 66% | 61% | 65% |
| CPN1 | rs10883437 | 49% | 38% | 45% | 40% | 52% |
| CYP2E1 | rs28969387 | 3% | 0% | 2% | 1% | 0% |
| DDX60L | rs2710833 | 13% | 15% | 15% | 15% | 17% |
| EFCAB4B | rs887304 | 24% | 19% | 19% | 15% | 17% |
| EHBP1L1 | rs6591182 | 41% | 66% | 63% | 43% | 55% |
| ENPP1 | rs1044498 | 37% | 42% | 36% | 29% | 19% |
| ERLIN1 | rs2862954 | 29% | 14% | 26% | 25% | 48% |
| FDFT1 | rs2645424 | 37% | 38% | 61% | 47% | 40% |
| GCKR | rs780094 | 38% | 22% | 35% | 39% | 36% |
| GCKR | rs1260326 | 39% | 24% | 34% | 38% | 35% |
| GCLC | rs17883901 | 6% | 0% | 5% | 8% | 6% |
| HERPUD2 | rs343062 | 38% | 38% | 31% | 35% | 38% |
| HSD17B13 | rs6834314 | 85% | 79% | 77% | 75% | 76% |
| IL28B | rs12979860 | N/A | N/A | 83% | 34% | 65% |
| IL6 | rs1800795 | 15% | 17% | 22% | 19% | 21% |
| IRS1 | rs1801278 | 0% | 0% | 4% | 5% | 4% |
| LCP1 | rs7324845 | 86% | 81% | 81% | 83% | 87% |
| LEPR | rs1137100 | 18% | 25% | 19% | 39% | 17% |
| LEPR | rs1137101 | 46% | 52% | 53% | 41% | 39% |
| LEPR | rs6700896 | 31% | 61% | 59% | 46% | 37% |
| LPIN1 | rs13412852 | 74% | 77% | 71% | 75% | 74% |
| LPPR4 | rs12743824 | 47% | 35% | 43% | 46% | 44% |
| LYPLAL1 | rs12137855 | 87% | 86% | 86% | 84% | 79% |
| MTTP | rs3816873 | 20% | 18% | 31% | 22% | 29% |
| MTTP | rs1800591 | 82% | 81% | 76% | 78% | 71% |
| MTTP | rs1800804 | 18% | 21% | 24% | 22% | 29% |
| NCAN | rs2228603 | 6% | 4% | 3% | 5% | 3% |
| NNMT | rs694539 | 26% | 28% | 16% | 24% | 18% |
| NR1I2 | rs2461823 | 33% | 57% | 43% | 39% | 64% |
| PARVB | rs6006611 | 56% | 58% | 48% | 43% | 41% |
| PDGFA | rs343064 | 37% | 34% | 30% | 36% | 38% |
| PEMT | rs7946 | 37% | 46% | 51% | 46% | 56% |
| PNPLA3 | rs2896019 | 41% | 17% | 25% | 28% | 28% |
| PNPLA3 | rs738409 | 71% | 88% | 81% | 28% | 36% |
| PNPLA3 | rs6006460 | 100% | 95% | 98% | 97% | 100% |
| PP1R3B | rs2126259 | 88% | 88% | 86% | 89% | 80% |
| PPARA | rs1800234 | 99% | 100% | 98% | 98% | 97% |
| PPARGC1A | rs8192678 | 20% | 25% | 27% | 29% | 29% |
| PPP1R3B | rs4240624 | 85% | 88% | 86% | 90% | 82% |
| PXR | rs7643645 | 35% | 41% | 32% | 32% | 38% |
| PZP | rs6487679 | 21% | 12% | 18% | 18% | 16% |
| SAMM50 | rs2143571 | 44% | 25% | 28% | 32% | 30% |
| SAMM50 | rs738491 | 46% | 31% | 33% | 40% | 33% |
| SAMM50 | rs3761472 | 38% | 17% | 21% | 25% | 21% |
| SLC27A5 | rs56225452 | 16% | 25% | 21% | 16% | 17% |
| SLC2A1 | rs2229682 | 19% | 15% | 19% | 14% | 25% |
| SLC38A8 | rs11864146 | N/A | N/A | N/A | 17% | 18% |
| SOD2 | rs4880 | 55% | 48% | 47% | 37% | 49% |
| STAT3 | rs6503695 | 31% | 32% | 33% | 33% | 19% |
| TCF7L2 | rs7903146 | 18% | 28% | 20% | 22% | 31% |
| TLR4 | rs4986791 | 5% | 4% | 3% | 3% | 6% |
| TRIB1 | rs2954021 | 42% | 50% | 51% | 44% | 43% |
| UCP3 | rs11235972 | 81% | 92% | 83% | 80% | 85% |
| UCP3 | rs1800849 | 18% | 8% | 16% | 20% | 15% |
| ZP4 | rs2499604 | 58% | 60% | 53% | 53% | 45% |
|  |  |  |  |  |  |  |
| N/A = Bad Signal; General Population and Puerto Rican Population from 1,000 Genomes Project | | | | | | |
